# Krüppel homolog 1 and E93 mediate Juvenile hormone regulation of metamorphosis in the common bed bug, *Cimex lectularius*

**DOI:** 10.1038/srep26092

**Published:** 2016-05-17

**Authors:** Hemant Gujar, Subba Reddy Palli

**Affiliations:** 1 Department of Entomology, University of Kentucky, Lexington, KY 40546-0091, USA

## Abstract

The common bed bug is an obligate hematophagous parasite of humans. We studied the regulation of molting and metamorphosis in bed bugs with a goal to identify key players involved. qRT-PCR studies on the expression of genes known to be involved in molting and metamorphosis showed high levels of *Krüppel homolog 1* [Kr-h1, a transcription factor that plays key roles in juvenile hormone (JH) action] mRNA in the penultimate nymphal stage (N4). However, low levels of *Kr-h1* mRNA were detected in the fifth and last nymphal stage (N5). Knockdown of Kr-h1 in N4 resulted in a precocious development of adult structures. Kr-h1 maintains the immature stage by suppressing E93 (early ecdysone response gene) in N4. E93 expression increases during the N5 in the absence of Kr-h1 and promotes the development of adult structures. Knockdown of E93 in N5 results in the formation of supernumerary nymphs. The role of JH in the suppression of adult structures through interaction with Kr-h1 and E93 was also studied by the topical application of JH analog, methoprene, to N5. Methoprene induced Kr-h1 and suppressed E93 and induced formation of the supernumerary nymph. These data show interactions between Kr-h1, E93 and JH in the regulation of metamorphosis in the bed bugs.

The resurgence of bed bugs in recent years in the USA and other countries around the world have brought bed bugs in the limelight again^1^. They are known to cause an allergic reaction in people, the severity of which varies from a mild rash to psychological disorder or anemia^2^,^3^,^4^. They also cause anxiety, impaired quality of life and psychosocial functioning and suicide in extreme cases^5^,^6^. They have been shown to be potential vectors of *Trypanosoma cruzi*^7^ that causes Chagas fever. It has been suggested that they might be a possible vector of methicillin-resistant *Staphylococcus aureus* and vancomycin-resistant *Enterococcus faecium*^8^.

Bed bugs go through five nymphal stages prior to becoming an adult. Nutrition is the major driver of molting and metamorphosis since bed bugs undergo molting or metamorphosis only after a blood meal. Juvenile hormones (JH) are sesquiterpenoids that are synthesized in the corpora allata^9^,^10^. JH functions through its receptor, Methoprene-tolerant protein (Met) and steroid receptor co-activator (SRC) or Cycle (CYC)^11^,^12^. Met is a member of the basic helix–loop–helix Per-ARNT-Sim (bHLH–PAS) gene family^13^. Krüppel homolog 1 (Kr-h1)^14^, hairy^12^ and trypsin^15^ are among the genes that are directly induced by JH. Kr-h1 is a zinc finger domain containing transcription factor and plays an important role in JH regulation of molting, metamorphosis and reproduction in insects^16^,^17^. Ecdysteroids are steroid hormones and regulate molting, metamorphosis and reproduction. 20-hydroxy ecdysone (20E) is the most active form of this hormone. 20E functions through a heterodimer of nuclear receptors, ecdysone receptor (EcR) and ultraspiracle (USP). The 20E-EcR-USP complex directly regulates expression early genes coding for transcription factors including E75, E74, E93 and Broad-Complex (*BR-C*). These proteins, in turn, regulate expression of early-late and late genes including nuclear receptors HR3 and HR4. E93 is a helix-turn-helix transcription factor containing a Pip-squeak motif. E93 was shown to be involved in regulation of metamorphosis and programmed cell death^18^.

Hormonal regulation of molting and metamorphosis has been well studied in many insects including some hemipteran insects. In *Blattella germanica* and *Rhodnius prolixus* Kr-h1 functions as an antimetamorphic factor^16^,^19^. In these insects, Kr-h1 levels drop during the final nymphal stage, which allows development of adult structures. E93 is highly expressed during the last nymphal stage and promotes nymph to adult transition^20^. E93 cross-talks with the JH pathway by downregulating Kr-h1 and *BR-C* expression during the last nymphal stage of *B. germanica*. Knockdown of E93 during the pupal stage of *Tribolium castaneum* leads to the development of supernumerary pupae^20^. In *Pyrrhocoris apterus*, Met and Kr-h1 but not BR-C are involved in antimetamorphic action^19^. Knockdown of these genes causes the development of adult color pattern, wings and genitalia. BR-C is a pupal specifier in holometabolous insects but specifies the immature stage in the direct developing hemimetabolous insects such as *Oncopeltus fasciatus.* In this insect, *BR-C* disappears during adult development^21^. Knockdown of *BR-C* in *O. fasciatus* causes the premature appearance of adult characteristics. Bed bug biology is unique; these insects can stay alive without a blood meal for months. However, initiation of molting and metamorphosis in bed bug, *C. lectularius* requires blood feeding^22^. In this paper, we report on the identification of key players that regulate molting and metamorphosis in the bed bugs.

## Results

### Expression of genes involved in molting and metamorphosis

**Figure 1:**
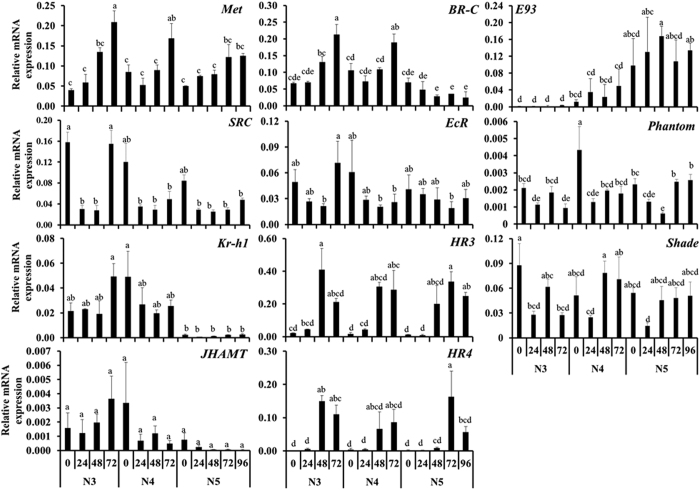
Expression profiles of developmental genes in *Cimex lectularius* nymphs. RNA from third (N3), fourth (N4) and fifth (N5) nymphal stages were collected at 24 hr intervals beginning at the time of feeding until they enter next stage. mRNA levels were determined using qRT-PCR and normalized using expression levels of ribosomal protein 8 (rpl8). Relative mRNA levels of genes regulating hormone pathways including Methoprene-tolerant protein (Met), Steroid receptor co-activator (SRC), Kruppel homologue 1 (Kr-h1), JH acid methyl transferase (JHAMT), Ecdysone receptor (EcR), Hormone receptor 3 (HR3), Hormone receptor 4 (HR4), Ecdysone-induced protein 93 F (E93), Phantom, Shade, and Broad-Complex (*BR-C*) are shown. Data shown are mean + SD (n = 3). (Alphabets represent significance at 95% CI).

Expression of genes known to be involved in molting and metamorphosis in other insects was determined during the third (N3), fourth (N4, penultimate) and fifth (N5, final) nymphal stages. Homologs of genes known to regulate molting and metamorphosis in other insects were identified in the bed bug and qRT-PCR primers were designed. The mRNA levels of these genes were quantified in insects collected at 24 hr intervals beginning at blood feeding until they molt to the next stage. Expression of JH receptor Methoprene-tolerant protein (Met) showed an increase after blood feeding until they molt to the next stage (Fig. 1). Higher levels of steroid receptor co-activator (SRC) mRNA were detected soon after feeding and then the mRNA levels decrease by 24–48 hr after feeding (Fig. 1). The SRC mRNA levels increase again and higher levels of this mRNA were detected at 72 hr after feeding. The Kr-h1 mRNA levels did not show significant changes during N3 and N4, however during N5, the Kr-h1 mRNA levels decreased by 16-fold when compared to their levels in N3 and N4 (Fig. 1). JHAMT (JH acid methyl transferase, an enzyme involved in JH biosynthesis) mRNA were detected during N3 and N4 but decreased to undetectable levels during N5 (Fig. 1). Broad-Complex (BR-C) mRNA levels increased from 0 hr to 72 hr after feeding in N3 and N4. In contrast, BR-C mRNA levels decreased from 0 hr to 96 hr after feeding in N5 (Fig. 1).

Ecdysone receptor (EcR) mRNA levels did not show significant differences among the stages tested suggesting that EcR gene is expressed most of the times during N3, N4 and N5. The mRNA levels of ecdysone delayed-early genes, hormone receptor 3 (HR3) and hormone receptor 4 (HR4) increased during each molt (Fig. 1). E93 mRNA was not detected during N3; the E93 mRNA levels started to increase at 24 hr after feeding during N4 and reached the maximum levels after a molt to N5 and these higher levels were maintained throughout N5 (Fig. 1). The mRNA of phantom and shade (the enzymes involved in ecdysteroid biosynthesis) were detected in N3, N4 and N5 and did not show significant differences among the stages tested (Fig. 1).

**Figure 2:**
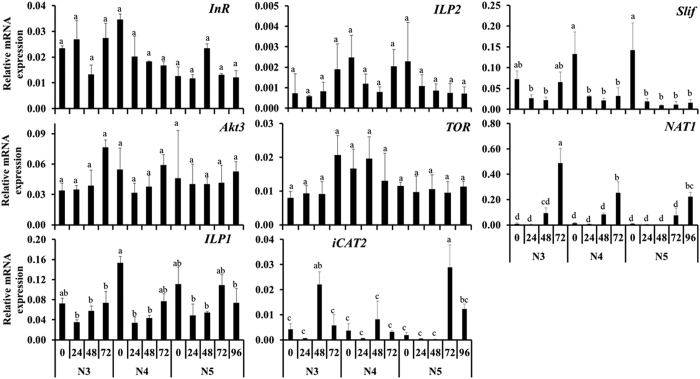
Relative mRNA levels of genes regulating nutrition pathway. RNA from third (N3), fourth (N4) and fifth (N5) nymphal stages were collected at 24 hr intervals beginning at the time of feeding until they enter next stage. mRNA levels were determined using qRT-PCR and normalized using expression levels of ribosomal protein 8 (rpl8). The relative mRNA levels of Insulin Receptor (InR), RAC-gamma serine/threonine-protein kinase (Akt3), Insulin-like peptide 1 (ILP1), Insulin-like peptide 2 (ILP2), Target of Rapamycin (TOR), insect cationic amino acid transporter 2 (iCAT2), Slimfast (Slif), and probable sodium-coupled neutral amino acid transporter 6 (NAT-1) are shown. Data shown are mean + SD (n = 3). (Alphabets represent significance at 95% CI).

Insulin receptor (InR), protein kinase B (Akt3), insulin-like peptide 1 (ILP1), insulin-like peptide 2 (ILP2) and target of rapamycin (TOR) mRNAs were detected during N3, N4 and N5 stages and did not show significant differences among the stages tested (Fig. 2). Cationic amino acid transporter iCAT2 expression showed a significant increase at 48 hr after a blood meal in N3 as well as at 72 and 96 hr after a blood meal in N5 (Fig. 2). Similarly, Slimfast (Slif), another cationic amino-acid transporter showed an increase in expression at 0 and 72 hr after a blood meal in N3 and soon after a blood meal in both N4 and N5. Whereas the Na (+)-coupled neutral amino acid transporter 6 (NAT1) showed a significant increase in expression prior to molting to N4, N5 and adult (Fig. 2).

Expression pattern of these key genes involved in JH and 20E action suggest that these two hormones play important roles in molting and metamorphosis of *C. lectularius.* Decrease in the expression of Kr-h1 and increase in the expression of E93 during N5 stage suggest that these two genes may play important roles in cross-talk between JH and 20E in the regulation of metamorphosis of *C. lectularius.* Therefore, we concentrated on determining the function of these two genes in the regulation of *C. lectularius* metamorphosis.

### Role of Juvenile hormone in regulation of metamorphosis

**Figure 3:**
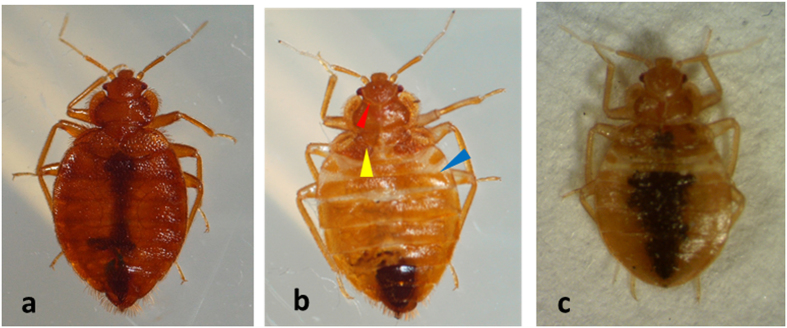
Methoprene induces a supernumerary molt. 10 μg of methoprene in cyclohexane was topically applied on the abdomen of N5 after blood feeding. Methoprene application was repeated every alternating day until molt. The control insects were applied with cyclohexane, these insects developed into adults (**a**). 18% of methoprene treated insects molted to N6 (**b**). 82% mortality was observed in methoprene treated insects. The N6 showed the presence of nymphal characters including the presence of first three less sclerotized segments at the anterior end of the abdomen (blue arrowhead), lighter sclerotization of the cuticle as compared to the adult, presence of ecdysial lines towards the head (red arrowhead) and partially developed wing pads (yellow arrowhead). The experiment was repeated three times and similar phenotypes shown in photographs have been observed in all experiments. Untreated N5 is shown in Fig. 3C for comparison.

To study the role of JH in the prevention of metamorphosis in the bed bugs, ten μg of JH analog, methoprene was applied on the abdomen of blood fed N5. An equal volume of cyclohexane was applied to control insects. Eighty-five percent of control cyclohexane treated insects molted into adults (Table 1, Fig. 3a). Methoprene application to N5 induced development of supernumerary nymphs (Fig. 3b). Eighteen percent of methoprene treated nymphs molted into the supernumerary nymphal stage and the rest of them died (Table 1). When compared to N5 is shown in Fig. 3c and adult shown in Fig. 3a, the supernumerary nymphs showed nymphal characters including lighter sclerotization of the cuticle especially the first three segments in the anterior region of the abdomen (blue arrowhead in Fig. 3b), the presence of ecdysial lines on the head (red arrowhead in Fig. 3b) and partially developed wing pads (yellow arrowhead in Fig. 3b).

### Kr-h1 plays a key role in JH regulation of metamorphosis

**Figure 4:**
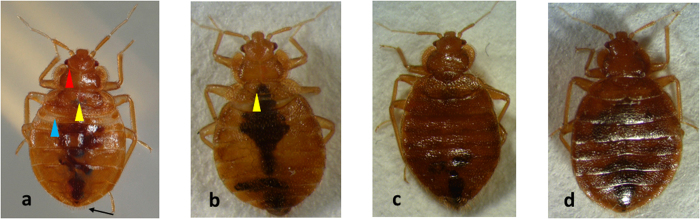
RNA interference (RNAi)-mediated knockdown of Kr-h1 in N4. Kr-h1 dsRNA was injected into fourth instar nymphs. The insects were incubated for four days before blood feeding. malE injected bugs were used as a control. 100% of the control bugs molted into fifth instar nymphs (**a**). 31.11% of dsKr-h1 injected bugs molted into a precocious adult (**b**), rest of them molted to N5. Control adults male (**c**) and female (**d**) are also shown for comparison. The precociously developed insects showed the development of wing pads (yellow arrowhead). Wing pads were fused in fifth instar nymphs. Three less sclerotized segments in the anterior region of the abdomen, which is normally seen in fifth instar nymph (blue arrowhead in Fig. 4a) are absent in the preciously developed adults. Ecdysial lines (red arrowhead in Fig. 4a) which are a characteristic feature of nymphs were also absent in the precociously developed adult and control adults. The experiment was repeated three times.

To determine the role of Kr-h1 in the regulation of metamorphosis, we prepared dsRNA targeting Kr-h1 and injected it into N4. The control insects injected with *malE* dsRNA molted into N5 (Fig. 4a) and showed typical characters of N5 consisting of first three less sclerotized segments at the anterior region of the abdomen (blue arrow head), lighter sclerotization of the cuticle, presence of ecdysial lines on the head (red arrowhead) and fused wing pads forming a concave line (yellow arrowhead). In contrast, 31% of the Kr-h1 dsRNA injected insects developed precociously into adults exhibiting external features including developed wing pads (Fig. 4b, yellow arrowheads), absence of first three less sclerotized abdominal segments, absence of ecdysial lines, and darker sclerotization of the cuticle as compared to that in control N5 (Table 2 and Fig. 4b). External morphology of adult male (Fig. 4c) and female (Fig. 4d) are shown for comparison.

**Figure 5:**
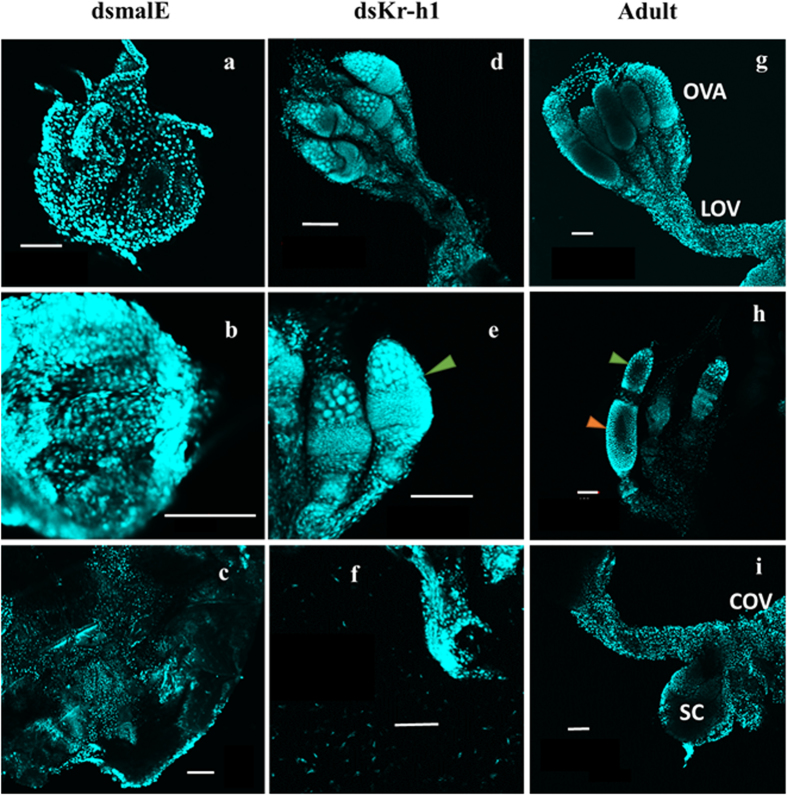
Precocious development of ovaries in Kr-h1 knockdown insects. RNAi-mediated knockdown of Kr-h1 was carried out in N4. Control insects injected with malE dsRNA molted into fifth instar nymphs. Ovary development was reduced in control N5 (**a–c**). Ovaries (**d–f**) form Kr-h1 dsRNA injected insects showed the precocious development of ovaries (**d–f**). These ovaries showed well-developed ovarioles (**e**) where the germarium (green arrowhead) is detected. Lateral oviduct and common oviduct are also developed (**f**). These structures including the germarium (green arrowhead) and oocytes (red arrowhead) are well developed in control adult (**g–i**). OVA, ovarioles; LOV, lateral oviduct; COV, common oviduct and SC, seminal conceptacle. Bar represents 100 μm.

malE and Kr-h1 dsRNA injected nymphs were dissected after molting to the next stage and the development of reproductive organs was recorded using a confocal microscope. The reproductive organs were not well developed in the control insets (Fig. 5a–c) when compared to those in Kr-h1 dsRNA injected insects (Fig. 5d–f). In control insects injected with malE dsRNA, the ovaries are smaller in size (Fig. 5a) as compared to the Kr-h1 dsRNA injected insects (Fig. 5d). The germarium or the vitellarium did not develop well in the control insects (Fig. 5b). The control insects did not show the presence of oviduct or seminal conceptacle (Fig. 5c). In contrast, Kr-h1 dsRNA injected showed developed ovaries (Fig. 5d–f). The ovaries are larger in size, germarium is clearly visible (Fig. 5e green arrowhead), lateral and common oviducts are developed (Fig. 5f). The ovaries dissected from adults are shown in (Fig. 5g–i) for comparison. The ovaries dissected from newly emerged adults are larger in size, the germarium is well developed (green arrowhead) and some of them showed the presence of vitellarium (orange arrowhead) (Fig. 5h). The lateral and common oviducts and seminal conceptacle are well developed in these ovaries (Fig. 5i).

**Figure 6:**
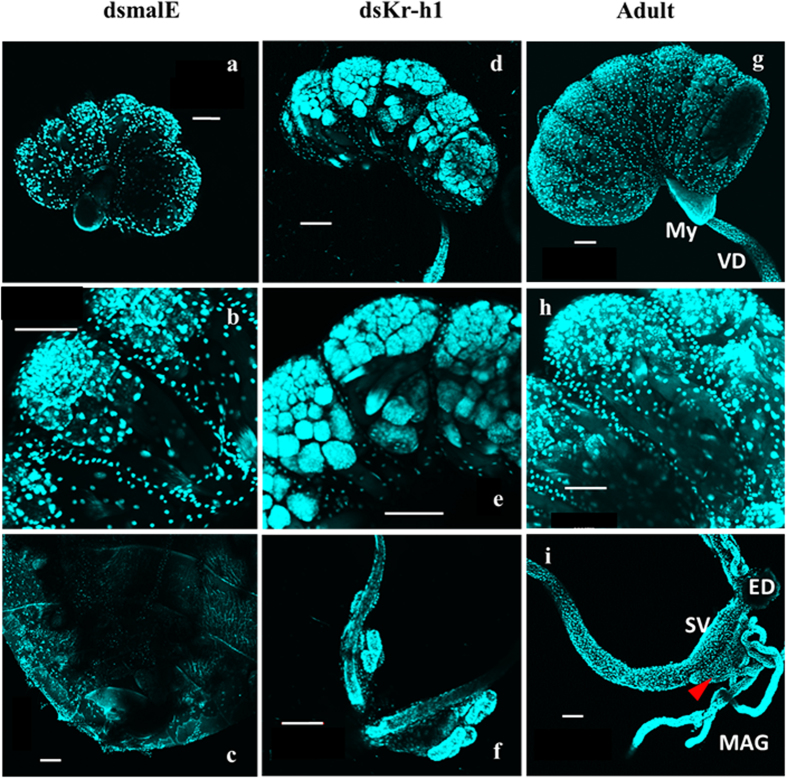
Precocious development of testis in Kr-h1 knockdown insects. RNAi-mediated knock down of Kr-h1 was carried out in N4. Control insects injected with malE dsRNA were dissected to observe the development of testis (**a–c**). Kr-h1 knockdown insects showed the development of male reproductive organ (**d–f**). Vas-deference (VD), seminal vesicle (SV), male accessory gland reservoir (Red arrow head) and male accessory glands (MAG) were observed in kr-h1 knockdown males. Male reproductive organs dissected from control adults are shown in (**g–i**) for comparison. Bar represents 100 μm.

The N4 insects injected with malE dsRNA molted into N5 and contained testis and mycetome (Fig. 6a). Figure 6b shows an enlarged view of a testicular lobe. However, the vas-deference, seminal vesicle, ejaculatory duct, male accessory gland reservoir and male accessory glands were absent in these insects (Fig. 6a and c). In contrast, Kr-h1 dsRNA injected nymphs showed well-developed testis (Fig. 6d,e), vas-deferens, seminal vesicles, male accessory gland reservoirs and male accessory glands (Fig. 6f). Ejaculatory pump and aedeagus were not clearly defined as seen in the control adult males. Reproductive system in control adults is well developed and showed seven testicular lobes, mycetome, seminal vesicle, ejaculatory duct, male accessory gland reservoir and male accessory gland (Fig. 6g–i).

### Role of E93 in regulation of metamorphosis

**Figure 7:**
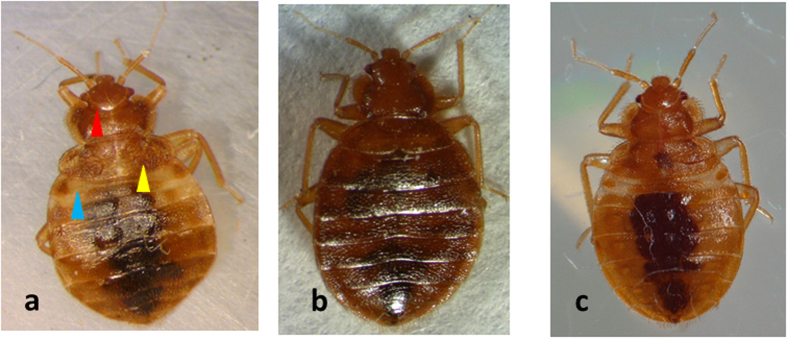
RNA interference (RNAi) mediated knockdown of E93 in N5. E93 dsRNA was injected into N5, *GFP* was used as a control. The control insects metamorphosed into adults (**b**). Whereas 50% of E93 injected insects molted into N6 (**a**). N6 showed first three less sclerotized abdominal segments (blue arrowhead) in the anterior region of the abdomen, partially developed wing pads (yellow arrowhead), ecdysial lines on the head (red arrowhead) and lighter sclerotization of the cuticle. 25% mortality was observed in this group, whereas 25% developed into adults. Control N5 is shown for comparison (**c**). The experiment was repeated three times with similar results.

RNAi-mediated knockdown of E93 was carried out in the N5. About 50% of E93 dsRNA injected insects molted into supernumerary nymphs, 25% developed into adults and the rest of the 25% died (Table 2). The supernumerary nymphs showed nymphal characters including the presence of first three less sclerotized segments at the anterior region of the abdomen (blue arrowhead in Fig. 7a), lighter sclerotization of the cuticle, underdeveloped wing pads (yellow arrowhead in Fig. 7a) and the presence of ecdysial lines on the head (red arrowhead, Fig. 7a). All control N5 injected with *GFP* dsRNA developed into adults (Fig. 7b). Fifth instar nymph is shown in Fig. 7c for comparison.

### Cross-talk between Kr-h1 and E93

**Figure 8:**
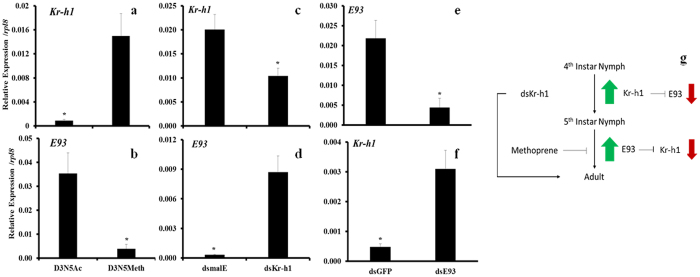
Interaction of Kr-h1 and E93 pathway in the control of molting and metamorphosis. After blood feeding of N5, 10 μg of methoprene was topically applied every day. RNA was extracted on the third day at 6 hours after methoprene application. qRT-PCR analysis of RNA isolated from these insects showed an increase in Kr-h1 mRNA levels (**a**) and suppression of E93 mRNA levels (**b**). One μg of Kr-h1 or malE dsRNA was injected into N4. The insects were fed after days and total RNA was extracted two days after feeding. Knockdown of Kr-h1 in N4 (**c**) caused a decrease in Kr-h1 mRNA levels and an increase in *E93* mRNA levels (**d**). Knockdown of E93 in N5 (**c**) caused a decrease in E93 mRNA levels (**e**) and an increase in Kr-h1 mRNA levels (**f**). Proposed model for cross-talk between Kr-h1 and E93 is shown in Figure (**g**).

To study the cross-talk between Kr-h1 and E93, we quantified mRNA levels of Kr-h1, E93 in insects injected with Kr-h1 or E93 dsRNA or treated with methoprene. Application of methoprene to day 3 N5 induced Kr-h1 mRNA levels by about 15-fold when compared to its expression in the control insects treated with acetone (Fig. 7a). In contrast, application of methoprene resulted in a seven-fold reduction in E93 mRNA levels when compared to its expression in control insects treated with acetone (Fig. 8b). Injection of Kr-h1 dsRNA caused 50% knockdown in Kr-h1 mRNA levels in N4 (Fig. 8c) and the knockdown in Kr-h1 resulted in 15-fold increase in E93 mRNA levels when compared to its expression in control insects injected with malE dsRNA (Fig. 8d). Injection of E93 dsRNA into N5 caused >80% knockdown in the expression of this gene (Fig. 8e) and a six-fold increase in Kr-h1 mRNA levels, when compared to its expression in control insects, injected with *GFP* dsRNA (Fig. 8f). These results suggest that Kr-h1 and E93 regulate each other and both of them are involved in JH suppression of metamorphosis (Fig. 8g).

## Discussion

Bed bug populations and problems caused by these insects are increasing in the USA and around the world. One of the reasons attributed to the resurgence of bed bugs is the development of insecticide resistance in these insects making them difficult to control using currently available insecticides^23^,^24^. New insecticides with a novel mode of action are urgently needed. The studies included in this paper have been conducted to understand the hormonal regulation of molting and metamorphosis in bed bugs with a goal to identify key genes involved in regulation of these processes.

Hormonal regulation of molting and metamorphosis was investigated in both hemimetabolous and holometabolous insects. In these insects JH and ecdysteroids play important roles in the regulation of these processes. Ecdysteroid titers peak before each molt or metamorphosis^25^. In hemimetabolous insects, JH titers are high in the immature stages but fall during the last instar stage^26^. Similarly, in holometabolous insect *Bombyx mori* JH titers fall during the last instar larvae but increase again before entering the pupae stage^27^. mRNA expression levels of Kr-h1 a JH-inducible gene has been shown to correlate well with the JH titers^16^. Kr-h1 mRNA expression profiles have been studied in hemimetabolous and holometabolous insects which include *Pyrrhocoris apterus*^19^,^28^, *Blattella germanica*^16^, *Tribolium castaneum*^29^, *Bombyx mori*^30^ and *Manduca sexta*^31^.

qRT-PCR studies on the expression levels of homologs of genes identified as key players in JH and 20E biosynthesis and action suggested that these two hormones regulate molting and metamorphosis in bed bugs. Significant reduction in the expression of Kr-h1 and increase in the expression of E93 during N5 stage suggested that these two genes might mediate cross-talk between JH and 20E action in regulation of molting and metamorphosis. The interaction between the two genes was confirmed by topical application of JH analog, methoprene, to fifth instar nymphs. Topical application of methoprene to fifth instar nymphs induced Kr-h1 and suppressed E93 expression while inducing a supernumerary molt. Knockdown of E93 in the fifth instar stage also induced Kr-h1 expression while inducing a supernumerary molt. E93 was induced in insects injected with Kr-h1 dsRNA. These data suggest that the presence of JH and Kr-h1 during N1-N4 promote nymphal molt and prevent metamorphosis. The function of Kr-h1 as a repressor of adult characteristics was more evident when its knockdown in the fourth instar stage resulted in the precocious development of ovaries and testis.

Injection of dsRNA targeting two regions of Met gene in *C. lectularius* resulted in 60–80% reduction in Met mRNA levels (Fig. S1). However, no detectable phenotype was observed. This may be because very low levels of Met protein remained in the RNAi insects is sufficient for its function. In silkworm, *Bombyx mori* knockdown of CYP15C1 and JHAMT (which are involved in JH synthesis pathway) results in the precocious development of adults^32^. Met and Kr-h1 knockdown also induce development of adult characters in holometabolous insect *Tribolium castaneum*^29^,^33^. In other hemimetabolous insects including *Blattella germanica, Pyrrhocoris apterus* and *Rhodnius prolixus* blocking JH action by knocking down Met and Kr-h1 results in the precocious development of adults from the penultimate stage nymphs^16^,^19^. Met knockdown in *B. germanica* during the penultimate stage causes precocious adult development. Whereas, its knockdown during the final nymphal stage causes developmental defects in the adults which include shortened wings and decrease in the expression of EcR, RXR, E75 and ILP-1^34^. In *B. germanica,* E93 defines nymph to adult molt^31^. E93 expression increases in the last stage nymphs and E93 induces the formation of adult characteristics. Similarly, in holometabolous insects E93 expression increases in the pupal stage and promotes adult development. Knockdown of E93 in the pupae prevents the formation of adults and results in the formation of the second pupa^20^.

The research reported here showed that Kr-h1 and E93 regulate molting and metamorphosis in *C. lectularius*. Based on our data and data reported from other hemimetabolous and holometabolous insects, we conclude that higher levels of JH during the penultimate nymphal stage induce Kr-h1 expression and suppress E93 expression to prevent metamorphosis. Whereas, a decrease in JH titers allows the expression of E93 and promotes the development of adult structures. Thus, the function of Kr-h1 and E93 in JH and 20E regulation of molting and metamorphosis is conserved throughout the hemimetabolous and holometabolous insects. However, molecular mechanisms of the cross-talk between Kr-h1 and E93 in transduction of JH signals that regulate molting and metamorphosis as well as the function of 20E in these interactions remain unknown and will be the focus of future studies.

## Methods

### Insects

#### *Cimex lectularius* NY-1 colony was used in this study

The insects were collected from an infested apartment in Plainview, New York in April, 2007. Insects were maintained at 26.7 °C, 65 ± 5% RH and a photoperiod of 14: 10 hr (L: D). Insects were maintained on defibrinated rabbit blood (at 37 °C) by the method developed by Montes *et al*.^35^. Blood was purchased from Quad Five Company. The nymphs were identified on the basis of size and morphology of the wing pads. The N5 wing pads fuse together forming a concave structure, whereas N4 show a straight line in between wing pads.

### Gene identification

#### De novo assembly of 454 sequences was performed

The files used in the assembly are as follows: NCBI Accession number 1) SRX028107; 2) SRX013985; 3) SRX013984; 4) EST *C. lectularius* (#7131) University of Kentucky CIN-1 strain (SRR3084449). All the contigs and singletons thus obtained were analyzed using Blast2go. BLASTX was then performed against NCBI NR database. Blast2go software was used to predict the function of assembled genes. GO ID, Enzyme ID and Interpro accession numbers were obtained for all the sequences. Maker annotated genes from the i5k project were also used for identification of some genes.

### RNA Isolation, cDNA Synthesis, and Quantitative Real-Time PCR (qRT-PCR)

Total RNA was isolated from three insects for each replicate using the TRI Reagent (Molecular Research Center Inc., Cincinnati, OH). The RNA was treated with DNase I (Ambion Inc., Austin, TX). cDNA was synthesized using Promega kit (Promega, Madison, WI). qRT-PCR was performed using Applied Biosystems Step One Plus TM (Life Technologies™ Real-Time PCR System, Carlsbad, CA). FastStart SYBR Green Master mix (Roche Diagnostics, Indianapolis, IN) and 2 μl of 10 μM primers were used in a ten μl qRT-PCR reaction. Primers used are shown in Table 1S. The mRNA levels were normalized using the internal control RPL8 (ribosomal protein L8).

### Double-stranded RNA synthesis and Injection

Fragments of genes coding for select genes were PCR amplified using the primers reported in Table 1S, and these DNA fragments were used to prepare dsRNA, as described by MEGA script RNAi synthesis Kit (Ambion Inc., Austin, TX). Newly molted N4 and N5 were anesthetized with ethyl ether vapor for 2 min and lined on a glass slide covered with double-sided tape. About one μg (0.1 μl) of dsRNAs were injected into the ventral side between the first and second abdominal segment using Nanojet an injection needle made using the needle puller (Idaho Technology, Salt Lake City, Utah). The dsRNA prepared using a fragment of *Escherichia coli* maltase gene (*malE*) or green fluorescent protein (*GFP*) was used as a control. Injected nymphs were removed from the slide after recovery and kept in an incubator for four days before feeding them with rabbit blood.

### Methoprene treatment

10 μg Methoprene in cyclohexane was applied on the abdomen of blood fed N5. Methoprene application was repeated on alternating days. The Same volume of cyclohexane was applied to control insects. The bugs were then kept in an incubator and allowed to molt.

### Light and Confocal Microscopy

Insect pictures were taken using DinoCapture2.0 software under white light. The tissues were dissected in 0.01 M phosphate buffer saline and fixed in 4% paraformaldehyde. Then the fixed tissues were rinsed with PBS and stained with DAPI. Pictures were then taken using confocal microscope under illumination with light at 405 nm wavelength.

### Statistical Analysis

Statistical analysis was performed using Statistix software. One way ANOVA was performed for comparison of expression data in different stages of the insect. Post hoc test consists of Tukey HSD. The level of significance was set at P = 0.05. Student t-test (unpaired t-test) was performed for comparing significance in knockdown and induction of gene expression at 95% confidence.

## Additional Information

**How to cite this article**: Gujar, H. and Palli, S. R. Krüppel homolog 1 and E93 mediate Juvenile hormone regulation of metamorphosis in the common bed bug, *Cimex lectularius. Sci. Rep.* **6**, 26092; doi: 10.1038/srep26092 (2016).

## Supplementary Material

Supplementary Information

## Figures and Tables

**Table 1:** Effect of methoprene application on metamorphosis.

Percent	Mortality	Supernumerary nymph	Adult
Cyclohexane	15	0	85
Methoprene	82	18	0

**Table 2:** Effect of Kr-h1 and E93 knockdown on metamorphosis.

Gene	Knockdown in 4th instar nymph			Knockdown in 5th instar nymph		
	Mortality (%)	N5 (%)	Precocious adults	Mortality (%)	Adult (%)	Supernumerary nymphs
malE	0	100	N5 nymphs	0	100	Adult
E93	–	–	–	25	25	50
Kr-h1	2	67	31	–	–	–
