# Supplementary material for: Krüppel homolog 1 and E93 mediate Juvenile hormone regulation of metamorphosis in the common bed bug, Cimex lectularius
Source: Sci Rep. 2016 May 17;6:26092. doi: 10.1038/srep26092 (PMC4869114; doi:10.1038/srep26092)
Supplement: Supplementary Information [file srep26092-s1.pdf]

Supplementary data

Krüppel homolog 1 and E93 mediate Juvenile hormone regulation of metamorphosis in the common bed bug, *Cimex lectularius*

Hemant Gujar and Subba Reddy Palli

Table 1S: Primers used in the studies reported in the MS

	dsRNA primers*	Forward Primer	Reverse Primer
1	Met	CCAAGCTCGACCAGAGAGATAAG	AGCTGCTCCGTGCTTGTAT
2	Kr-h1	TTCGGATAACAACCTCCGCTTGGA	GGTCCACAATCGGAGTCACACA
3	Br	TGAGGCAAAGGTCTCTTTCGTCGT	TTCATCGGCATTGCATTGTCCTGG
4	E93	GACGTCATCACTAACACGATAA	GAACACTCATCTCTCCTCTTTG
**S.No.**	**qRT-PCR primers**	**Forward Primer**	**Reverse Primer**
1	Met	TCGTCCCTGGTCACAGAAACGAAA	ACTGCGGTTGATCGCTCTTCGATA
2	SRC	ATGAGCTCACTTTCCGTCAAGCCT	GGTCGCAGATGCTTGCTGTTCTTT
3	Kr-h1	ACGCTTTGGCGTACTGAATAACGG	TTTCGGGATCGCCGATTTCTGTCT
4	BR-C	ATCGCCAAGGGAAGACATGGAAGA	TGGAGTCTTCCCGCTCATTGTGAA
5	EcR	AAGGGAAGACTTATCACCGCCCAA	TGTAATGGTAACCTGAAGCCCGGT
6	HR3	TTGACCCTGTCAACGAGACAAGC	ATCACATGCGAGGGTTGCAAGG
7	HR4	AAGAAACCTCGATGATCTCTTAG	GAGTTCAAGGTAGAAGGGTAATC
8	E93	AAGCGTTGGTGAGTAAAGAG	GTTGGAAGGAGGATAGGAAATAG
9	InR	AGACGGCAGATGAGCTTGCTAAGT	AATGGAGGCTGTTCAAGGGTCTCA
10	Akt3	GGGCTGTGGAATCAATTCAGCCAT	GCAGCCATTTCATCGGACGTGTTA
11	TOR	GGACAATGGCCTCAAGTGTATGAAGC	ACCAATCGACCGACAAAGGTACGA
12	ILP1	AACGTGGCTACAACATGGCGTTTC	ATCCCTCGCTGCAGTACGATTCAA
13	ILP2	GTGGCCGGACTTTAACGAAGATGT	ATCCATTGCAAATGACTCGGAGCG
14	Phantom	TAATCGGCATTGTAGGGCCTGTCA	CTTTCAGGTAGCGCAACAAACGGT
15	Shade	TCCAAATCGCTTCTTCCCTCACGA	GCGAACAACAAAGCCCTTGACGAT
16	iCAT2	GTCCACTCGAAGTACAAGAC	CGATAGACATGAGATCGACTAAG
17	Slif	GCTGGAGATGGTCTTCTATTC	CCTGAAATCAATCCTGAGAGTAT
18	NAT1	GCTCTAGGTTCCTACAACAAAT	AACCGCCAATCCTGAATAAA
19	JHAMT	GCTCTTCGTTTGGGATTCT	CTTCTCAGAGGTGAAAGTCTAC

*All dsRNA primers contain T7 sequences on the 5’ end

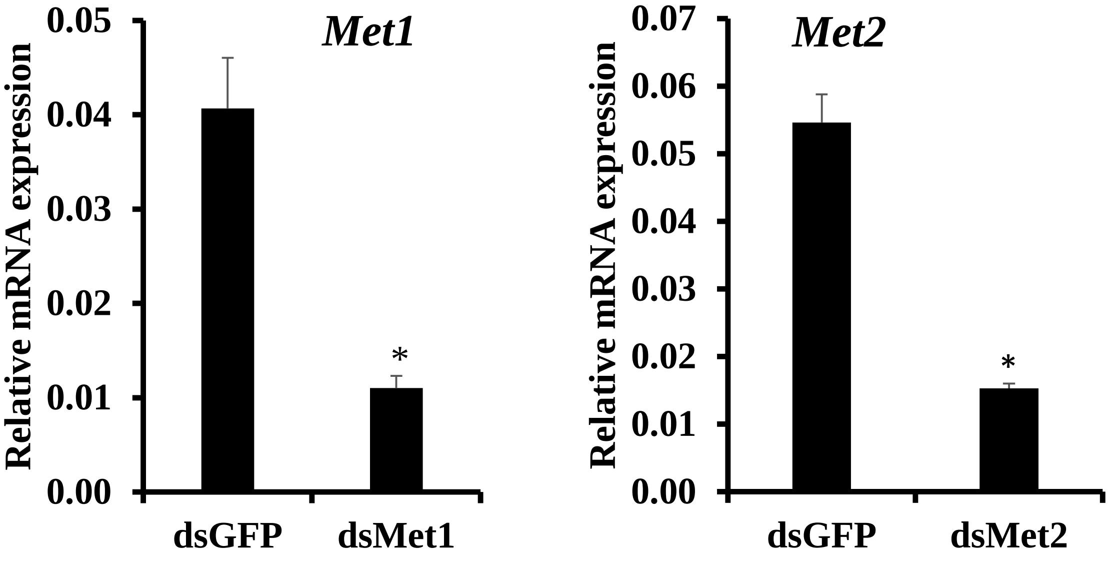

Fig. S1: Knockdown of Methoprene-tolerant protein was performed by injecting Met dsRNA into newly molted fourth instar nymphs. The insects were incubated for five days before feeding. Total RNA was collected two days after feeding, and knockdown was observed. Two different RNAs targeting different regions of Met were used. . Each bar represents Mean + SD of three biological replicates (Star represents the level of significance with Student t-test at $p \leq 0.05$).

Table 2S: Phenotype observed with Met mRNA knockdown in fourth instar nymphs.

Percent	injection in 4th instar nymph		
Gene	Mortality	N5	Phenotype
malE	0	100	N5
Met	0	100	N5
